# The Calcineurin Inhibitor-Sparing (CIS) Trial - individualised calcineurin-inhibitor treatment by immunomonitoring in renal allograft recipients: protocol for a randomised controlled trial

**DOI:** 10.1186/1745-6215-15-489

**Published:** 2014-12-13

**Authors:** Claudia Sommerer, Matthias Schaier, Christian Morath, Vedat Schwenger, Geraldine Rauch, Thomas Giese, Martin Zeier

**Affiliations:** Department of Nephrology, University Hospital Heidelberg, Im Neuenheimer Feld 162, D-69120 Heidelberg, Germany; Institute of Medical Biometry and Informatics, University of Heidelberg, Im Neuenheimer Feld 305, D-69120 Heidelberg, Germany; Department of Immunology, University Hospital Heidelberg, Im Neuenheimer Feld 305, D-69120 Heidelberg, Germany

**Keywords:** Immunomonitoring, Renal transplantation, Calcineurin inhibitor, Ciclosporin A, Gene expression, CIS study, Minimisation, Immunosuppression, NFAT

## Abstract

**Background:**

Adequate monitoring tools are required to optimise the immunosuppressive therapy of an individual patient. Particularly, in calcineurin inhibitors, as critical dose drugs with a narrow therapeutic range, the optimal monitoring strategies are discussed in terms of safety and efficacy. Nevertheless, no pharmacokinetic monitoring markers reflect the biological activity of the drug. A new quantitative analysis of gene expression was employed to directly measure the functional effects of calcineurin inhibition: the transcriptional activities of the nuclear factor of activated T-cell (NFAT)-regulated genes in the peripheral blood.

**Methods/Design:**

The CIS study is a randomised prospective controlled trial, comparing a ciclosporin A (CsA)-based immunosuppressive regimen monitored by CsA trough levels to a CsA-based immunosuppressive regimen monitored by residual NFAT-regulated gene expression. Pulse wave velocity as an accepted surrogate marker of the cardiovascular risk is assessed in both study groups. Our hypothesis is that an individualised CsA therapy monitored by residual NFAT-regulated gene expression results in a significantly lower cardiovascular risk compared to CsA therapy monitored by CsA trough levels.

**Discussion:**

There is a lack of evidence in individualising standard immunosuppression in renal allograft recipients. The CIS study will consider the feasibility of individualised ciclosporin A immunosuppression by pharmacodynamic monitoring and evaluate the opportunity to reduce cardiovascular risk while maintaining sufficient immunosuppression.

**Trial registration:**

EudraCT identifier 2011-003547-21, registration date 18 July 2011

https://www.clinicaltrialsregister.eu

## Background

### Current challenges for individualised immunosuppression

Calcineurin inhibitors (CNI) represent the most widely used immunosuppressive agents in kidney transplantation. More than 80% of all renal allograft recipients are on CNI therapy [1]. Since the introduction of ciclosporin A (CsA) in the early 1980s and tacrolimus (Tac) in the 1990s, acute rejection rates have improved remarkably. The current one-year survival rate of a renal allograft exceeds 90% [2]. In recent decades, the overall renal allograft survival has not improved as expected, indicating a shift of graft failure from early acute rejection to side effects of long-term immunosuppression and chronic rejection. In addition, death with a functioning graft is an important aspect in the long-term follow-up after successful renal transplantation. Cardiovascular (CV) disease is the leading cause of death in kidney transplant recipients (KTRs), with a 3.5% to 5% annual risk of fatal or non-fatal CV events, much higher than in the general population despite adjustment for traditional risk factors [3, 4].

Undesirable side effects of CNIs, such as nephrotoxicity, metabolic deterioration, aggravation of CV risk factors, as well as the CNI contribution to the development of chronic allograft dysfunction, have come into the focus [5]. There are many efforts to reduce or withdraw CNIs in order to decrease renal complications as well as other side effects. However, insufficient immunosuppression with an increased risk of rejection should be avoided [6, 7].

In particular, the development of donor-specific antibodies (DSA) and humoral rejection are the focus of recent renal transplant research [8]. Concerning the avoidance of relevant side effects, one of the investigational strategies is to minimise CNIs combined with adequate mycophenolic acid (MPA) exposure [9–11]. This strategy might result in decreased cardiovascular risk, improved renal allograft function and prolonged patient as well as allograft survival.

Adequate monitoring tools are required to optimise the immunosuppressive therapy of an allograft recipient. Particularly in CNIs, as critical dose drugs with a narrow therapeutic range, optimal monitoring strategies are discussed in terms of safety and efficacy [12]. Nevertheless, no pharmacokinetic (PK) monitoring markers reflect the biological activity of the drug, namely suppression of alloreactive immune responses.

### Pharmacodynamic monitoring as a tool to optimise immunosuppression

Detailed knowledge of the drug-specific mode of action is required to establish and introduce a pharmacodynamic (PD) monitoring assay. The proposed immunosuppressive mode of CsA is the inhibition of the phosphatase activity of calcineurin after binding of the corresponding CNI-immunophilin complexes [13, 14]. The main substrate of calcineurin in T-cells is the phosphorylated transcription factor ‘nuclear factor of activated T-cells’ (NFAT). NFAT dephosphorylation by calcineurin is required for the nuclear translocation and subsequent transcriptional activation of several key genes of T-cell activation, such as interleukin-2 (IL-2), tumour necrosis factor alpha (TNF-α), and interferon gamma (IFN-γ) [15].

Recently, a new quantitative analysis of gene expression was employed to measure directly the functional effects of calcineurin inhibition: the transcriptional activities of NFAT-regulated genes in the peripheral blood [16, 17]. Several evaluations affirmed this approach as a useful tool with the potential to individualise CNI therapy. A high interindividual variability of residual NFAT-regulated gene expression in patients with corresponding CsA or Tac doses has been observed, which confirms various degrees of immunosuppression and T-cell activation. On the other hand, intraindividual variability of residual NFAT-regulated gene expression was low in repetitive measurements in one single patient with a stable CNI dose. This pharmacodynamic monitoring tool might have the potential for precise individual dosing of CNIs [16, 18, 19].

To date, several evaluations and preliminary studies have been published concerning residual NFAT-regulated gene expression as a specific CNI monitoring tool in clinical practice. NFAT-regulated gene expression in CsA treatment has been evaluated in solid organ recipients, such as kidney, liver and heart in adult and paediatric patient cohorts [18–27]. All these studies show a cross-sectional or prospective observational design, except for one prospective interventional case-control study [19]. In this study, 20 stable renal allograft recipients were compared to 20 matched controls to analyse the feasibility of stepwise adaption of CsA dosage by residual NFAT-regulated gene expression. Tapering of CsA resulted in an increase of residual NFAT-regulated gene expression from 6% (1 to 17) to 21% (7 to 32); and resulted in an improved renal allograft function and blood pressure [19].

Comprehensive data on CsA monitoring by NFAT-regulated gene expression are available, therefore, a prospective randomised interventional trial was established using CsA as a long-term worldwide-used immunosuppression. The Calcineurin Inhibitor-Sparing (CIS) trial is the first randomised controlled study in stable renal allograft recipients to evaluate this immunomonitoring method for tapering of immunosuppression. The rationale and design of the trial are introduced here.

### Research aim and objectives

The CIS trial assesses for the first time in a randomised prospective study, the improvement of the CV risk in stable renal allograft recipients on a CsA regimen by monitoring of standard CsA trough levels (C0s), compared to the novel approach by residual NFAT-regulated gene expression. It takes into account both the need to prevent allograft rejection and to optimise CV risk in an established immunosuppressive regimen.

## Methods/Design

### Study design

**Figure 1:**
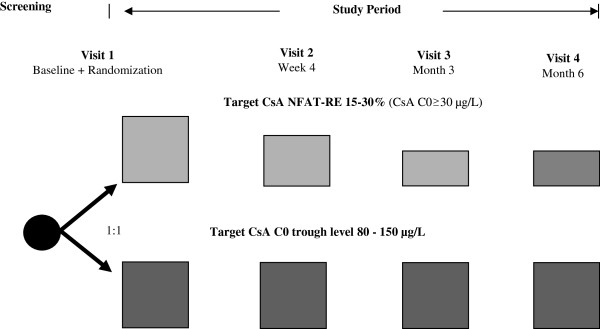
**Design of the Calcineurin Inhibitor-Sparing (CIS) trial.** CsA, ciclosporin; CNI, calcineurin inhibitor; MPA, mycophenolic acid.

The CIS trial (RCHD-1004, protocol version 1.2, 23 December 2013) is a prospective, randomised, parallel-grouped, confirmatory study (Figure 1). This trial is a single-centre study performed at the Renal Clinic Heidelberg (Department of Nephrology, University Hospital Heidelberg). Stable renal allograft recipients on CsA treatment are randomised, to be monitored and adapted either by CsA C0s or residual expression of NFAT-regulated genes. The study period is six months.

### Selection of the primary endpoint

The primary objective of this trial is to show that a CsA-based immunosuppressive regimen monitored by residual NFAT-regulated gene expression results in a reduced CV risk, compared to monitoring by standard CsA C0s in renal allograft recipients. The CV risk is assessed by the change of arterial stiffness measured by pulse wave velocity (PWV) from baseline to Month 6.

The PWV is an established sensitive marker for the assessment of CV risk, confirmed in a large number of clinical studies including end-stage renal disease (ESRD) population, older or transplanted patients [28, 29]. In a former study by our group, PWV was significantly lower in renal allograft recipients with a CNI-free regimen in comparison to CsA-treated patients [30].

### Outcome measures

The change in the arterial stiffness assessed by PWV from baseline (Visit 1) to Month 6 (Visit 3) is the primary efficacy objective of the study. Carotid-femoral PWV pressure as well as central blood pressure will be assessed using a validated SphygmoCor device from AtCor Medical, Sydney, Australia. The assessment of PWV is performed blinded to drug treatment, results of CsA C0 or residual NFAT-regulated gene expression. Measurement of PWV as well as peripheral and central blood pressure is performed at randomisation (Visit 1), Month 3 (Visit 3), and Month 6 (Visit 4).

Secondary efficacy and safety objectives are given in Table 1. One secondary objective of the CIS trial is to evaluate the two treatment groups with respect to a composite efficacy failure endpoint comprising biopsy-proven acute rejection (BPAR), graft loss or death at the end of the study, consistent with recommendations by the European Medicines Agency (EMEA) [31]. Additional secondary objectives include individual components of this composite failure endpoint.

**Table 1:** **Objectives of the Calcineurin Inhibitor-Sparing (CIS) trial**

Primary objective	To evaluate a CsA-based immunosuppressive regimen monitored by residual NFAT-regulated gene expression concerning reduction in cardiovascular risk assessed by the change in pulse wave velocity from baseline to six-month follow-up, compared to a CsA-based regimen monitored by CsA trough levels in renal allograft recipients
Secondary efficacy objectives	To evaluate, in treatment group:
	- a composite endpoint of biopsy-proven acute rejection (BPAR), graft loss, death and loss to follow-up at Month 6
	- Incidence of BPAR, graft loss, loss to follow-up or death at Month 6
	- S-creatinine and cystatin C at Month 6.
	- Renal allograft function (eGFR calculated by MDRD, Nankivell and Cockroft-Gault formulae) at Month 6
	- Evolution of renal function (S-creatinine) over time by slope analysis
	- Creatinine slope (1/serum creatinine versus time) including the treatment period between baseline and Month 6
Secondary safety objectives	To evaluate, in treatment group:
	- Incidence and severity of adverse events (AEs) and serious adverse events (SAEs)
	- Incidence of AEs leading to discontinuation from the study
	- Incidence of major cardiovascular events (myocardial infarction, apoplexy, peripheral arterial occlusive disease)
	- Pulse wave velocity, aortic pulse pressure, aortic systolic pressure, aortic augmentation index, ejection duration, heart rate variability
	- Changes in blood pressure (central and peripheral pulse pressure, systolic and diastolic, mean arterial blood pressure)
	- Changes in antihypertensive medication (number of antihypertensives)
	- Changes in lipids (cholesterol, LDL-, HDL-cholesterol, triglyceride) and lipid-lowering drugs
	- Changes in glucose levels, HbA1c and antidiabetic therapy
	- Changes in cardiovascular risk (for example Framingham score)
	- Changes in CsA-induced side effects (for example hypertrichosis, gingival overgrowth)
	- Incidence and severity of infections
	- Incidence and severity of malignancies
	- Changes in the quality of life assessed by the ESRD SCL™ questionnaire and SF12 questionnaire
Exploratory objectives	To explore the incidence of DSA in treatment group, and in relation to acute rejection, in a subset of patients at participating centres

BPAR, biopsy-proven acute rejection; CsA, ciclosporin A; DSA, donor-specific antibody; eGFR, estimated glomerular filtration rate; ESRD, end-stage renal disease; HDL, high-density lipoprotein; LDL, low-density lipoprotein; MDRD, Modification of Diet in Renal Disease; SCL, symptom checklist; SF12, Short-Form 12.

For the analysis of laboratory data, venous blood will be drawn prior to medication intake and the blood probe will be analysed in the centre’s local laboratory at each visit. The renal function will be assessed by estimated glomerular filtration (EGF) rate, serum creatinine levels at each study visit, cystatin C at baseline and end of study, and the evolution of renal function (S-creatinine) over time by slope analysis.

Safety objectives include standard assessments of adverse events (AEs) and serious adverse events (SAEs), and specific objectives related to CV events such as hypertension, hyperlipidaemia, and diabetes. In addition, at each study visit, specific objectives that are of concern in renal allograft recipients are assessed, including comorbidities such as malignancies, viral infections and CsA-induced side effects at each study visit. Vital signs will be documented at each study visit and a physical examination carried out at baseline and end of study. In all suspected rejection episodes, an allograft biopsy should be performed prior to or at the latest within 24 hours after the initiation of anti-rejection therapy. Biopsies will be read and interpreted by an independent pathologist blinded to drug treatment according to the updated Banff criteria.

The allograft will be presumed to be lost on the day the patient starts dialysis and is not able to subsequently be removed from dialysis or if the patient undergoes a graft nephrectomy.

Information will be captured at each study visit on CV disease, including angina pectoris that leads to hospitalisation or to intervention, myocardial infarction (segment elevation myocardial infarction (STEMI), non-STEMI), stroke (defined as brain ischaemia due to thrombosis, embolism, or systemic hypoperfusion) and peripheral arterial occlusive disease. At baseline and end of study, the patients’ CV risk will be calculated using the Framingham score [32].

All patients will be screened for gingival hypertrophy and hypertrichosis at baseline and end of study. Health-related quality of life assessment will be collected by the ESRD symptom checklist (SCL) questionnaire and Short-Form 12 (SF-12) scale [33, 34] at baseline and end of study. Both questionnaires will be self-completed by the patients.

Exploratory objectives include evaluation of the incidence of DSA in the treatment group. The clinical significance of DSAs - specifically whether or not they contribute to antibody-mediated rejection - is currently a topic under discussion concerning optimising long-term renal allograft function [35].

Residual NFAT-regulated gene expression will be assessed at every visit in both treatment groups. This will provide additional data on the optimal range of residual NFAT-regulated gene expression in renal allograft recipients, even with respect to allograft function and rejection risk.

A time schedule of enrolment, intervention, assessments and visits is shown in Table 2.

**Table 2:** **Schedule of enrolment, interventions, and assessments**

	STUDY PERIOD			
PHASE	Baseline	Post-allocation		End of study
Visit	1	2	3	4
Day	0			
Month		1 ± 7d	3 ± 14d	6 ± 14d
**ENROLMENT**				
Eligibility screen	x			
Inclusion/exclusion	x			
Randomisation (allocation)	x			
**INTERVENTIONS**				
Intervention A (control group): adaption of CsA according to CsA C0	x	x	x	
Intervention B (investigational group): adaption of CsA according to NFAT-expression	x	x	x	
**ASSESSMENTS**				
Demography	x			
General medical history	x			
Transplantation information	x			
Physical examination	x			x
Vital signs	x	x	x	x
Study medication check		x	x	x
Laboratory test:				
Hematocrit/Biochemistry				
basic program		x	x	
extended program	x			x
Urinanalysis	x			x
CsA C0 and C2 levels	x	x	x	x
NFAT-regulated gene expression	x	x	x	x
Pulse wave velocity	x		x	x
Rejection episodes	as necessary			
Renal biopsy	as necessary			
Adverse events	as necessary			
Severe adverse events	as necessary			
Comments	as necessary			
Concomitant therapy	as necessary			
Immunosuppressive therapy	x	x	x	x
Framingham score	x			x
Quality of life assessment	x			x

C0, CsA trough level; C2, two-hour level; CsA, ciclosporin A; NFAT, nuclear factor of activated T-cell.

### Study population

The study population comprises 55 stable adult renal allograft recipients. The study has broad eligibility criteria and enrols CsA-treated patients with stable renal allograft function. Deceased as well as living donor recipients will be included. The eligibility criteria exclude patients with already significantly deteriorating renal allograft function or a history of chronic active rejection. Specific key inclusion and exclusion criteria are shown in Table 3.

**Table 3:** **Key inclusion and exclusion criteria for the Calcineurin Inhibitor-Sparing (CIS) trial**

Key inclusion criteria	Key exclusion criteria
- Male or female patients ≥18 years old.	- Patients with a history of acute rejection classified > BANFF II, chronic active antibody-mediated rejection or chronic T-cell-mediated rejection.
- Recipients of deceased or living kidney transplants.	- Patients with an EC-MPS dose of <720 mg/d (MMF <1000 mg/d) and MPA-AUC <30 mg^*^h/L.
- Time after the last renal transplantation at least six months.	- Patients with symptoms of significant somatic or mental illness. Inability to cooperate or communicate with the investigator, who are unlikely to comply with the study requirements, or who are unable to give informed consent.
- Stable renal allograft function, defined as S-creatinine ≤3.5 mg/dL and Δ S-creatinine ≤30% during the last three months.	- Females of childbearing potential who are planning to become pregnant, who are pregnant or lactating, and/or who are unwilling to use effective means of contraception, unless
- Patients who are willing and able to participate in the study and from whom written informed consent has been obtained.	a. their career, lifestyle, or sexual orientation precludes intercourse with a male partner, or b. their partners have been sterilised by vasectomy or other means
	- Evidence of drug or alcohol abuse
	- Patients actively taking part in an interventional trial

AUC, area under the curve; EC-MPS, enteric-coated mycophenolate sodium; MMF, mycophenolate mofetil; MPA, mycophenolic acid.

### Patient selection, randomisation and study treatment

All renal allograft recipients of the outpatient clinic of the Department of Nephrology, University Hospital Heidelberg, Germany, will be screened according to inclusion and exclusion criteria. Patients will be enrolled consecutively. After informed consent has been signed, the patient participates in the study. Each patient is uniquely identified by a patient number. The investigator dispenses the patient numbers in chronological order.

Patients are randomised after providing written informed consent in a 1:1 ratio by a web-based randomisation tool using block randomisation with fixed block lengths (Randomisation In Treatment Arms (RITA) randomisation system (University of Lübeck, Germany)). In patients assigned to the standard group (control group), CsA is administered adapted to CsA C0s of 80 to 150 μg/L. In patients assigned to the investigational group (NFAT group), CsA is administered adapted to residual NFAT-regulated gene expression of 15 to 30%. For safety reasons, CsA C0 should be ≥30 μg/L in all patients.

Patients should stay within the target trough blood levels or residual NFAT-regulated gene expression throughout the study. The adaption of CsA dosage starts after randomization as soon as the results of CsA C0 (control group) or NFAT-regulated gene expression (NFAT group) are available, and at subsequent visits, if necessary. Dose adjustments are performed in the control group if CsA whole blood levels are outside the target range of CsA C0 80 to 150 g/L and in the NFAT group if NFAT-regulated gene expression is outside the range of 15 to 30% at the time of study visits (baseline, Month 1, Month 3 and Month 6). Adaption of CsA dose will be performed according to the regular clinical practice in approximately 20% steps. In the case of severe CNI toxicity, dose reductions below the target levels may be performed at the investigators’ discretion. If CsA is interrupted for safety-related considerations for more than four consecutive weeks or more than two episodes longer than two weeks, the patient should be withdrawn from the study.

### Concomitant immunosuppression

The immunosuppressive regimen used in the study is widely accepted in clinical practice. All patients receive CsA and MPA therapy, with or without steroids. MPA is administered in a standard dosage of enteric-coated mycophenolate sodium (EC-MPS) (Myfortic™) 180 to 720 mg twice a day (BID) or mycophenolate mofetil (MMF) (Cellcept™) 250 to 1,000 mg BID. MPA exposure will be monitored if daily EC-MPS dose is below 720 mg/d or if MMF dose is below 1,000 mg/d. In this case, PK monitoring of EC-MPS will include MPA C0, C1, C2, C3, C4 and C6; and MPA-area under the curve (AUC) will be calculated according to Sommerer *et al.* [36]. PK monitoring of MMF will include MPA C0, C0.5 and C2; and MPA-AUC will be calculated according to the Fixed-Dose Concentration-Controlled (FDCC) study [37]. Target MPA-AUC for both drugs is 45 mg^*^h/L with a minimum of 30 mg^*^h/L.

Corticosteroids are administered according to local centre practice.

### Roles and responsibilities

The CIS study was designed by a steering committee consisting of nephrologists specialised in renal transplantation, an immunologist and a statistician. The CIS study is an investigator-initiated trial; the study sponsor is the Renal Clinic Heidelberg, Germany (Department of Nephrology, University Hospital Heidelberg, Germany). Regulatory study approval is provided by the competent authority, the ‘German Regulatory Authority’ as well as by the local Ethics Committee. The study is conducted by the investigators and the local Study Centre at the Department of Nephrology, University Hospital Heidelberg. Data management and biometry will be provided by the Institute of Medical Biometry and Informatics, University Heidelberg, Germany. Pharmacovigilance and monitoring will be performed by representatives of the independent Clinical Study Centre, University Heidelberg, Germany.

### Ethical, regulatory and management considerations

The study protocol (version 1.2, 23 December 2013) has received ethical and governance approvals by the German Regulatory Authority (reference number 4038718) as well as by the local Ethics Committee (Ethics Committee, University Hospital Heidelberg, Germany; reference number AFmo-622/2012). The CIS study was registered in the European Union Drug Regulating Authorities Clinical Trials (EudraCT) database on 18 July 2011 (EudraCT Identifier 2011-003547-21). The study protocol follows the principles of good clinical practice (GCP) and the study is performed in compliance with the Declaration of Helsinki. Researchers collecting data will have appropriate research permissions, where required. Written informed consent will be taken from each participant prior to study enrolment by an experienced investigator. All data will be managed and stored with protection of data privacy.

Documentation as well as corrections, if necessary, will be done in accordance with GCP guidelines. All protocol-required information collected during the trial must be entered in the case report form (CRF). The completed CRFs will be sent to the Institute of Medical Biometry and Informatics, University Heidelberg, Germany. Data assessment will be performed twice by two independent persons to avoid incorrect entries. The completeness, validity and plausibility of data are examined by validating programs, which thereby generate queries. The investigator or the designated representatives are obliged to clarify or explain the queries. All changes from the original data are documented on audit files. Data will be maintained using the statistical SAS program, Version 9.0 or higher (SAS Institute Inc., Cary, NC, USA). Monitoring will be performed regularly by the independent Clinical Study Centre, University Hospital Heidelberg, to check the completeness of the contents of records and the CRFs, the adherence to the protocol and to GCP, the progress of enrolment, and to ensure that study drug is being stored, dispensed, and accounted for according to specifications. Patient insurance for compensation to those who suffer harm from trial participation is provided.

### Sample size calculation

The changes for PWV between baseline (Visit 1) and six-month follow-up (Visit 3) are calculated and compared between the groups. For the CsA standard group, the estimated change in PWV is close to 0. It is further assumed that the baseline values are similar for both groups. From study reports by Bahous *et al.* [29] and Seckinger *et al.* [30], the probable change from baseline to the six-month follow-up in the CsA low-dose group is estimated as δ = 1.3 m/s. Since some patients are expected to reveal treatment complications that might decrease the differences between the two treatment groups, the expected absolute effect considered for sample size calculation is δ = 1.2. The standard deviation is assumed to be σ = 1.5 ml/min. Similar standard deviations have been reported by Seckinger *et al*. [30] and Bahous *et al.* [29], in different comparison scenarios. However, as the absolute effect was conservatively estimated, the proposed assumptions are likely to yield a sample size that is large enough. With α = 0.05 and 1-β = 80%, n = 26 patients per group are required to demonstrate superior efficacy of the NFAT group compared to the CsA standard group in PWV when using a two-sided unpaired *t* test. Including a small drop-out rate of 5%, this results in a total number of 55 patients.

### Statistical analysis

Statistical analyses will be performed by an independent statistician. The aim of the study is to prove that the CsA therapy monitored by residual NFAT-regulated gene expression is superior to CsA therapy monitored by CsA C0s, by testing the following hypotheses: the null hypothesis is that the change in PWV between baseline and Month 6 is the same in both treatment arms. The alternative hypothesis is that the change in PWV between baseline and Month 6 is lower or higher in the NFAT group than in the control group.

Analysis of covariance (ANCOVA) will be applied with treatment, age, baseline PWV and eGFR as covariates. The primary analysis will be performed on the intention-to-treat (ITT) population. The treatment groups will be compared, using least-square means derived from the ANCOVA model. The two-sided significance level is given by 0.05. Using an ANCOVA model instead of the two-sided unpaired *t* test, which was used for sample size calculation, increases the strength of the study, as the adjustment for covariates leads to a reduction in variance.

Missing values will be replaced by the ‘last observation carried forward’ (LOCF) approach. All secondary variables will be analysed in an exploratory way. Event rates will be estimated using the Kaplan-Meier method to handle patients who discontinue the treatment prior to suffering sufficiently from an event. The two groups will be compared using the log-rank test. This procedure will be applied for the BPAR, graft loss, death, as well as the composite endpoint of treatment failure. The main analysis will be performed at the last patient’s last visit at Month 6. No interim analyses or design adaptations are planned.

### Reporting

The CIS trial results will be reported in concordance with the Consolidated Standards of Reporting Trials (CONSORT) checklist [38].

## Discussion

There is the need to optimise treatment with a well-established standard immunosuppressive, such as CNIs, since there is a lack of new agents significantly improving short- and long-term outcome in renal transplantation. Monitoring of immunosuppression by the specific biological effect provides the opportunity for individualised immunosuppression with potential benefit regarding patient morbidity and mortality, as well as long-term allograft function.

The strength of the CIS trial is that this is the first prospective randomised controlled trial exploring residual NFAT-regulated gene expression as a novel approach for the monitoring of CsA treatment, in comparison to the standard monitoring by CsA C0. In addition, CsA peak level will also be assessed as several transplant centres use this monitoring technique. PK and PD analyses will be performed uniquely of all enrolled patients, including controls; drug dosages will be applied according to predefined criteria. The feasibility of CsA treatment by monitoring of NFAT-regulated gene expression will be assessed.

The present study includes stable renal allograft recipients - a population with a great need for the optimisation of the immunosuppressive regimen with established drugs in order to improve long-term allograft and patient survival. In this patient cohort in particular, it could be shown that there is a high interindividual variability in residual NFAT-regulated gene expression despite CsA C0s within the normal range [18]. Reduction of CsA dosages guided by monitoring of NFAT-regulated gene expression might be possible in a majority of these patients [19]. In the CIS study, the benefits of PD monitoring on cardiovascular risk factors as well as the effects on renal allograft function will be assessed in detail. In addition, signs for insufficient immunosuppression will be carefully evaluated, including the explorative evaluation of the incidence of DSA in the treatment group.

Cardiovascular events are the main cause of morbidity and mortality in the long-term outcome of renal allograft recipients. They are also an important cause of death with a functioning graft. Aortic stiffness assessed by PWV has been proven as an independent predictive value for all-cause and CV mortality, CV disease, fatal and non-fatal coronary events and fatal strokes in patients with various levels of CV risk [28]. PWV is accepted as a marker of target organ damage by national and international guidelines, for example the European Society of Hypertension guidelines [39]. As shown in recent studies, aortic stiffness assessed by PWV is also an independent risk factor of CV events in the short- and long-term interval in renal allograft recipients [40, 41]. Future multi-centre studies including a greater number of patients will be designed to investigate the opportunity of NFAT monitoring with respect to hard endpoints, such as a combined endpoint consisting of BPAR, graft loss, loss to follow-up, and death as well as to other endpoints of interest, such as renal function.

The limitation of the study is that it is a single-centre study that includes 55 patients of one transplant centre. However, patient number is defined based on careful sample-size calculation including an adequate attrition rate. It is anticipated that the present study will indicate strategies to minimise patient attrition and missing data. Larger longitudinal multi-centre studies on PD CsA monitoring by residual NFAT-regulated gene expression assessing renal outcome will be performed, including the scientific and practical experiences of the CIS study.

## Conclusions

In conclusion, administration of CsA according to the biological effect, the residual NFAT-regulated gene expression, includes the opportunity to lower the CV risk and specific CsA-induced side effects in renal allograft recipients while maintaining adequate immunosuppression. The present study investigates this novel approach and will provide valuable information on the potential validity and feasibility of this particular PD monitoring method.

## Trial status

The study opened to recruitment in October 2013 and is expected to be complete by June 2014.

## Abbreviations

AE: adverse event
ANCOVA: analysis of covariance
AUC: area under the curve
BID: twice a day
BPAR: biopsy-proven acute rejection
C0: trough level
C2: two-hour level
CNI: calcineurin inhibitor
CRF: case report form
CsA: ciclosporin A
CV: cardiovascular
DSA: donor-specific antibody
EC-MPS: enteric-coated mycophenolate sodium
eGFR: estimated glomerular filtration rate
EMEA: European Medicines Agency
ESRD: end-stage renal disease
GCP: good clinical practice
IFN-γ: interferon gamma
IL-2: interleukin 2
ITT: intention-to-treat
KTRs: kidney transplant recipients
LOCF: last observation carried forward
MDRD: Modification of Diet in Renal Disease
MMF: mycophenolate mofetil
MPA: mycophenolic acid
NFAT: nuclear factor of activated T-cell
PD: pharmacodynamic
PK: pharmacokinetic
PWV: pulse wave velocity
SAE: serious adverse event
SCL: symptom checklist
SF-12: Short-Form 12
STEMI: segment elevation myocardial infarction
Tac: tacrolimus
TNF-α: tumour necrosis factor alpha.
